# Supplementary material for: Evaluation of the Pathogenic Potential of Escherichia coli Strains Isolated from Eye Infections
Source: Microorganisms. 2022 May 25;10(6):1084. doi: 10.3390/microorganisms10061084 (PMC9229993; doi:10.3390/microorganisms10061084)
Supplement: Supplementary file 1 [file microorganisms-10-01084-s001.zip › microorganisms-1715729-supplementary.pdf]

Table S1. Clinical details of patients with eye infections caused by *Escherichia coli* strains

Strain	Gender[a]	Age[b]	Date	Diagnosis	Sample	Eye[c]	Signs and symptoms
K-1	M	49 y	13/11/11	Keratitis	Corneal scraping	OD	Pain, grittiness, hyperemia, discharge, photophobia, epiphora and decreased vision
K-2	M	47 y	12/07/11	Keratitis	Corneal scraping	OD	Pain, epiphora, hyperemia, photophobia, and grittiness
K-3	F	-	24/04/06	Keratitis	Contact lenses	OS	-
K-4	F	86 y	15/12/15	Keratitis	Corneal scraping	OS	Hyperemia, discharge, and decreased vision
K-5	F	50 y	23/11/18	Keratitis	Corneal scraping	OS	Itchiness, pain, hyperemia, discharge, photophobia, grittiness, and decreased vision
K-6	M	76 y	11/12/17	Keratitis	Corneal scraping	OS	Itchiness, hyperemia, photophobia, grittiness, and decreased vision
C-1	F	50 y	02/10/06	Conjunctivitis	Conjunctival swab	OD	Nyctalopia, pain, epiphora, and photophobia
C-2	M	2 m	17/06/08	Neonatal Conjunctivitis	Conjunctival swab	OD	-
C-3a<br>C-3b	F	71 y	08/05/07	Conjunctivitis	Conjunctival swab	OS	Hyperemia, discharge, photophobia, and epiphora
C-4	F	72 y	18/06/07	Conjunctivitis	Conjunctival swab	OS	Dry eye and discharge
C-5	M	64 y	01/06/09	Conjunctivitis	Conjunctival swab	OS	Itchiness, hyperemia, and epiphora
C-6	F	62 y	12/03/14	Conjunctivitis	Conjunctival swab	OD	Itchiness, hyperemia, and discharge
C-7	F	-	12/06/19	Conjunctivitis	Conjunctival swab	OD	-
C-8	M	3 m	05/04/05	Neonatal Conjunctivitis	Conjunctival swab	OD	Hyperemia and discharge
C-9	M	3 m	19/04/05	Neonatal Conjunctivitis	Conjunctival swab	OD	-
C-10	M	58 d	12/12/11	Neonatal Conjunctivitis	Conjunctival swab	OD	Hyperemia, discharge, and epiphora
C-11	M	16 d	30/08/18	Neonatal Conjunctivitis	Conjunctival swab	OD	Hyperemia and discharge

Table S1. Cont.

Strain	Gender	Age	Date	Diagnosis	Sample	Eye	Signs and symptoms
C-12	M	5 m	10/01/11	Neonatal Conjunctivitis	Conjunctival swab	OD	Discharge
C-13	M	9 d	25/08/14	Neonatal Conjunctivitis	Conjunctival swab	OD	Discharge and hyperemia
C-14	M	2 m	30/01/06	Neonatal Conjunctivitis	Conjunctival swab	OD	Discharge
C-15	F	25 d	04/09/06	Neonatal Conjunctivitis	Conjunctival swab	OS	Discharge

[a,] M, male; F, female.

[b,] y, years; m, months; d, days.

[c,] OD: Right eye; OS: Left eye. All unknown data is represented by a "-"

Table S2. Antibiotic susceptibility profile of *Escherichia coli* strains isolated from eye infections [a]

Antibiotic and class	K-1	K-2	K-3	K-4	K-5	K-6	C-1	C-2	C-3a	C-3b	C-4	C-5	C-6	C-7	C-8	C-9	C-10	C-11	C-12	C-13	C-14	C-15
**Aminoglycosides**																						
· Amikacin	S	S	S	S	S	S	S	S	S	S	S	S	S	S	S	S	S	S	S	S	S	S
· Gentamicin	S	S	S	S	R	S	S	S	S	S	S	S	S	S	S	S	S	S	S	S	S	S
β-lactam																						
**Cephalosporins**																						
· Cefepime	S	S	S	S	S	S	S	S	S	S	S	S	S	S	S	S	S	S	S	S	S	S
· Cefoxitin	S	S	S	S	S	S	S	S	S	S	S	S	S	S	S	S	S	S	S	S	S	S
· Cefotaxime	S	S	S	S	S	S	S	S	S	S	S	S	S	S	S	S	S	S	S	S	S	S
· Ceftazidime	S	S	S	S	S	S	S	S	S	S	S	S	S	S	S	S	S	S	S	S	S	S
**Monobactam**																						
· Aztreonam	S	S	S	S	S	S	S	S	S	S	S	S	S	S	S	S	S	S	S	S	S	S
**Carbapenem**																						
· Imipenem	S	S	S	S	S	S	S	S	S	S	S	S	S	S	S	S	S	S	S	S	S	S
· Ertapenem	S	S	S	S	S	S	S	S	R	S	S	S	S	S	S	S	S	S	S	S	S	S
· Meropenem	S	S	S	S	S	S	S	S	S	S	S	S	S	S	S	S	S	S	S	S	S	S
**Fluoroquinolones**																						
· Ciprofloxacin	S	S	S	R	R	S	S	S	S	S	S	R	R	S	R	R	S	S	S	S	S	S
**Glycylcyclines**																						
· Tigecycline	S	S	S	S	S	S	S	S	S	S	S	S	S	S	S	S	S	S	S	S	S	S

[a], S, Sensitive; R, Resistant.
